# Patterns and predictors of readmission among sepsis survivors in a tertiary emergency department in Ethiopia

**DOI:** 10.1016/j.ijregi.2025.100808

**Published:** 2025-11-17

**Authors:** Meron H. Biza, Chernet T. Mengistie, Biruk T. Mengistie, Mikiyas G. Teferi, Tsion K. Admas, Nardos B. Feleke, Gadissa B. Tafa, Finot Debebe, Tigist Worku

**Affiliations:** 1 Emergency and Critical Care Medicine Department, College of Health Sciences, Addis Ababa University, Addis Ababa, Ethiopia; 2 School of Medicine, College of Health Sciences, Addis Ababa University, Addis Ababa, Ethiopia

**Keywords:** Sepsis readmission, Sepsis survivors, Hospital readmission, Ethiopia, Low- and middle-income countries (LMICs), Post-discharge care

## Abstract

**Objectives:**

Sepsis is a leading cause of critical illness worldwide. Survivors often suffer long-term physical, cognitive, and psychological impairments. Post-sepsis rehospitalization is common but poorly characterized in low-resource settings. We aimed to determine 30/90/180-day readmission rates and predictors after emergency sepsis in Addis Ababa.

**Methods:**

We conducted a retrospective cohort study of adult patients meeting Sepsis-3 criteria (infection with ≥2-point rise in Sequential Organ Failure Assessment [SOFA]) admitted to a tertiary Ethiopian emergency department from 2019-2021. A modified SOFA score (five parameters) was used to define organ dysfunction, as has been validated for resource-limited settings. We recorded patient demographics, comorbidities (including cardiovascular disease and malignancy), acute severity (modified SOFA, septic shock, intensive care unit admission), microbiology, antibiotic duration, and outcomes. Survivors were followed for 180 days for hospital readmissions at any site of suspected infection. Multivariable logistic regression identified factors independently associated with 180-day readmission.

**Results:**

Of 110 sepsis patients, 39 (35.5%) died during index admission. Among 71 survivors, 32 (45.1%) were readmitted within 180 days (78.1% within 30 days; 15.6% days 31-90; 6.3% days 91-180). Infectious diagnoses accounted for 71.9% of readmissions (often recurrent pneumonia or urinary tract infection). Median time to first readmission was 16 days (interquartile range 5-41). Multivariable analysis showed higher odds of 180-day readmission in younger patients (adjusted odds ratio [OR] 2.53 per decade decrease in age, 95% confidence interval 1.09-5.87, *P* = 0.029), and lower odds in those with non-metastatic malignancy (adjusted OR 0.15, 0.03-0.75, *P* = 0.027) or longer index hospitalization (adjusted OR 0.31 per 5-day increase, 0.11-0.89, *P* = 0.033).

**Conclusions:**

Nearly half of emergency sepsis survivors were readmitted within 180 days, predominantly within 30 days, and most readmissions were for infection. Predictors included younger age, malignancy status, and length of stay. These findings highlight the need for targeted discharge planning, antimicrobial stewardship, and early follow-up for high-risk patients in low-resource settings.

## Highlights

• Nearly half of emergency sepsis survivors were readmitted within 180 days.
• Most readmissions (78%) occurred within 30 days, primarily for infectious causes.
• Younger age, malignancy status, and shorter hospital stay predicted readmission.
• Readmission mortality was high (18.7%), emphasizing post-sepsis vulnerability.
• Findings underscore the need for structured discharge planning and early follow-up in low- and middle-income countries.

## Introduction

Sepsis imposes a substantial global health burden, with recent estimates suggesting 48.9 million incident cases and 11.0 million sepsis-related deaths (19.7% of global mortality) [1]. In sub-Saharan Africa, sepsis incidence and mortality are especially high, driven by endemic infections (human immunodeficiency viruses [HIV], tuberculosis) and limited critical care resources [2,3]. A systematic review of adult sepsis in sub-Saharan Africa (SSA) found in-hospital mortality of approximately 19% for sepsis and 39% for severe sepsis, and noted that sepsis in SSA is dominated by HIV and tuberculosis [2]. Similarly, an urban Malawian cohort reported high sepsis incidence and mortality (in-hospital mortality ∼24%) [4]. In Ethiopia, sepsis and septic shock among intensive care unit (ICU) admissions carry high mortality rates; for instance, one study reported an ICU mortality of 41.8% among sepsis/septic shock cases [5]. These data underscore that sepsis is a leading cause of death worldwide and particularly in SSA [1,4]. For perspective, sepsis is estimated to have accounted for about $20 billion (5.2% of U.S. hospital costs in 2011), highlighting its major economic burden [6]. These high mortality rates in SSA, together with constrained outpatient continuity and limited patient tracking in many settings, increase the likelihood that sepsis survivors will leave the hospital with unresolved illness or functional deficits that predispose them to rehospitalization; therefore, readmission is an especially relevant and understudied outcome in Ethiopia [2,7].

Beyond acute mortality, many survivors of sepsis face long-term sequelae, including cognitive impairment, neuromuscular weakness, and psychological disorders [6,8]. These impairments contribute to reduced quality of life and substantial healthcare utilization. In high-income countries (HICs), hospital readmission rates among sepsis survivors are high and comparable to those for congestive heart failure and pneumonia [8]. Meta-analyses report 30-day rehospitalization rates around 20-25%, with nearly half of survivors requiring readmission within 1 year [9,10]. Infection is the most common cause of readmission (∼70%), often involving recurrent or unresolved pneumonia/urinary tract infection [9]. Non-infectious readmissions include cardiovascular events, venous thromboembolism, and renal failure [10,11]. In HIC cohorts, early readmissions (occurring within 15 days) are common and often relate to the index illness, whereas later readmissions may reflect new complications or chronic conditions [8]. Survivors of sepsis thus contribute disproportionately to hospital readmissions and healthcare costs [8,12]. For example, older age, male sex, and multiple comorbidities have been linked to especially high rehospitalization risk among survivors [10]. These findings have led to suggestions (in high-income settings) for interventions like early outpatient follow-up and rehabilitation to reduce readmissions, though their applicability in low- and middle-income countries (LMICs) is uncertain.

The Sepsis-3 definition standardizes case identification, describing sepsis as life-threatening organ dysfunction arising from an abnormal, dysregulated host response to infection [6]. Operationally, this entails an acute increase of ≥2 points in the Sequential Organ Failure Assessment (SOFA) score [1,6]. Septic shock is a subset with circulatory and metabolic abnormalities (vasopressors plus lactate >2 mmol/L), carrying >40% hospital mortality [6]. In practice, full SOFA requires extensive labs. A modified SOFA (mSOFA), using fewer parameters, has been validated in resource-limited settings, showing comparable prognostic value [13]. For readmission analyses, common surveillance intervals are 30, 90, and 180 days post-discharge. These timeframes capture early and mid-term events and align with clinical practice metrics [9].

Several clinical factors have been linked to post-sepsis readmission. Older age, higher comorbidity burden (e.g., diabetes, chronic lung/kidney disease, malignancy, cardiovascular disease), ICU admission, and greater illness severity (including septic shock) have been associated with increased readmission risk [8,9]. Conversely, some data suggest that chronic organ dysfunction may be a stronger predictor than acute severity [10,12]. Prolonged initial hospital stay and longer antibiotic therapy are plausible modifiers: longer stays may allow more complete treatment of infection, while shorter antibiotic courses could lead to recurrence [14,15]. Blood culture positivity and targeted antimicrobial therapy could also influence outcomes, though data are limited [16].

Importantly, virtually all data on post-sepsis readmission derive from high-income settings: one recent review noted that such studies have largely been conducted only in countries with substantial follow-up infrastructure [17]. In contrast, continuity of care after discharge is much weaker in Ethiopia and similar low-resource settings. Lack of formal post-discharge follow-up, often due to patients’ financial or travel constraints, is a well-documented barrier to ongoing care [7]. For example, telephone monitoring after surgery in Ethiopian hospitals identified nearly half of post-discharge infections that routine care missed [7]. Most hospitals rely on paper records without linkage, so patients returning to care elsewhere go untracked. Indeed, underuse of electronic medical records in Ethiopian hospitals is known to undermine patient monitoring and data quality [18]. These system gaps make long-term monitoring of sepsis survivors difficult. Some interventions, for instance, scheduled outpatient follow-up, have reduced 30-day readmission risk by roughly 20% in other conditions (e.g., heart failure) [19]. In Ethiopia, tailored strategies might include enhanced discharge planning, patient education, and community follow-up to address the needs of high-risk survivors.

Given the rising incidence of sepsis and these challenges, we aimed to characterize the rates, causes, and predictors of hospital readmission among adult sepsis survivors in Ethiopia. Understanding these patterns is essential for improving post-sepsis care and reducing preventable hospitalizations in low-resource settings.

## Methods

### Study design and setting

We performed a hospital-based, retrospective cross-sectional chart review of adult patients admitted with sepsis to the adult emergency department (ED) of Tikur Anbessa Specialized Hospital (TASH), Addis Ababa, Ethiopia. TASH is a tertiary referral and teaching hospital with about 800 beds that serves approximately 370,000-400,000 patients annually. The adult ED receives an average of 1,200 admissions per month. The study period was from 1 July 2023 to 31 July 2024; data abstraction occurred following institutional approval.

### Participants, eligibility, and sampling

The source population comprised all adult patients (age >13 years at TASH) admitted to the adult ED during the study period. The study population included patients diagnosed with sepsis or septic shock during an index ED admission. Inclusion criteria were: age >13 years and a documented diagnosis of sepsis or septic shock during the index admission. Exclusion criteria were: leaving against medical advice during the index admission; transfer from another facility for the rehospitalization event; readmission for non-medical reasons; and known metastatic malignancy with sepsis. Convenience sampling was used: card numbers of all patients meeting the inclusion criteria were identified from the ED triage logbook, online registry, and patient charts, and eligible charts were retrieved sequentially until the planned sample was reached.

### Sample size

A single-proportion formula was used to estimate sample size with Zα/2 = 1.96 (95% confidence interval [CI]), *P* =0.31 (estimated 180-day readmission prevalence from a comparable LMIC study), and d = 0.05, yielding n₀ ≈ 328. Applying the finite population correction for an estimated annual sepsis admission population (N ≈ 120) produced an adjusted sample ≈ 89. Allowing ≈10% for incomplete charts, the final planned sample size was **98** records.

### Case definitions and outcomes

Sepsis was defined according to Sepsis-3 as suspected or confirmed infection with an acute increase in organ dysfunction (ΔSOFA ≥2) [6]. Since some SOFA components were inconsistently recorded, we applied a five-component mSOFA score validated for resource-limited settings [13,20], comprising respiratory (SpO₂/FiO₂ used as a surrogate when PaO₂ was unavailable) [21], cardiovascular (mean arterial pressure [MAP]/vasopressor requirement), central nervous system (Glasgow coma scale [GCS]), renal (serum creatinine or documented oliguria), and hepatic (scleral icterus/jaundice). Baseline organ function was assumed zero unless chronic dysfunction was documented. The primary outcome was hospital readmission to TASH within 180 days of discharge; secondary outcomes were in-hospital mortality during the index admission and index hospitalization length of stay (days).

### Data sources and collection procedures

Data were abstracted using a structured case report form adapted from published tools. Trained data collectors (ED/ICU nurses, residents, and laboratory staff) extracted information from ED triage logbooks, inpatient charts, the hospital health management information system, and referral documents. Collected variables included demographics, comorbidities (used to calculate the Charlson Comorbidity Index), mSOFA components, source of infection, culture results, ICU admission, discharge hemoglobin, acute kidney injury, length of stay, outcomes, and readmission details. The tool’s content validity was reviewed by intensivists and infectious disease clinicians.

### Data quality assurance

A pretest on five non-study charts was performed, and the CRF was revised accordingly. Data collectors received training, and the principal investigator supervised abstraction with daily spot checks. Completed forms were reviewed for missing or inconsistent entries; charts missing the primary outcome or essential exposure variables were excluded from relevant analyses.

### Statistical analysis

Data were entered and analyzed using IBM SPSS Statistics v26. Continuous variables are reported as medians with interquartile ranges (IQRs) and categorical variables as counts and percentages. Group comparisons used Mann–Whitney U tests for continuous variables and chi-square or Fisher’s exact tests for categorical variables, as appropriate. Univariable logistic regression screened candidate predictors of 180-day readmission; variables with *P* ≤0.20 and clinically important covariates (age, sex, and Charlson Comorbidity Index) were included in multivariable logistic regression models. Data on socioeconomic status, nutritional status, and post-discharge follow-up were not systematically available in medical records and were therefore not included as covariates in the regression models. Adjusted odds ratios (ORs) with 95% CIs are reported. Model fit was assessed with standard diagnostics. A two-sided *P* ≤0.05 was considered statistically significant. Missing data were handled by complete-case analysis; the extent of missingness is reported in Results.

### Missing data assessment

We evaluated the completeness of all key study variables and reported the proportion of missing observations in Table 1. Analyses were performed on available cases. As culture documentation was frequently missing (≈59%), but other variables were >90% complete, complete-case analysis was deemed appropriate. Missing data patterns likely reflected gaps in routine laboratory recording rather than selective exclusion.

**Table 1:** Completeness of key variables.

Variable	n missing	% missing	Comment
Age	0	0%	Complete
Sex	0	0%	Complete
Charlson comorbidity index	2	1.8%	Near-complete
Total modified Sequential Organ Failure Assessment score	3	2.7%	Near-complete
Culture result	65	59.1%	Limited laboratory documentation
Length of stay	0	0%	Complete
Intensive care unit admission	0	0%	Complete
Outcome (mortality/readmission)	0	0%	Complete

### Ethical considerations

Ethical approval was obtained from the Addis Ababa University College of Health Sciences, Department of Emergency and Critical Care Medicine Institutional Review Board (IRB approval no. AAU-CHS/MS/2024/201), and additional authorization was granted by the Addis Ababa Health Bureau. Since this study was a retrospective review of de-identified patient records, the IRB waived the requirement for individual informed consent. All procedures adhered to the ethical standards of the institutional and national research committees and to the Declaration of Helsinki (latest revision). Confidentiality was maintained by using coded study identifiers and secure, password-protected electronic storage accessible only to the research team.

## Results

### Cohort and baseline characteristics

A total of 110 patients meeting the sepsis definition were included. The overall median age was 53 years (IQR: 35-67); 58 (51.8%) were male. Thirty-nine patients (35.5%) died during the index admission, and 71 (64.5%) survived to discharge. Among the entire cohort, respiratory infections were the most frequent source (50%), followed by gastrointestinal (20%) and urinary (16.4%) infections. The median mSOFA score was 4 (IQR: 2-9), and the median Charlson Comorbidity Index was 3 (IQR: 0-13). Culture was positive in 15 patients (13.6%) (Table 2).

**Table 2:** Demography and clinical characteristics of sepsis patients at index admission.

Characteristics N(%)	Overall 110	Death 39 (35.5%)	Survived 71 (64.5%)
**Demographic data**			
Age, year median, interquartile range	53 (35-67)	60 (45-71)	48 (35-60)
**Sex n(%)**			
Male	58 (51.8%)	23 (58.9%)	35 (49.3%)
Female	52 (48.2%)	16 (41%)	36 (50.7%)
**Clinical data (Source of infection)**			
Respiratory	55 (50%)	27 (69.2%)	28 (40%)
Urinary	18 (16.4%)	3 (7.6%)	15 (21.4%)
Gastrointestinal	22 (20%)	5 (12.8%)	16 (22.8%)
Others	15 (13.6%)	4 (10.2%)	12 (17%)
**Comorbidities**			
Diabetes mellitus n(%)	23 (20.9%)	12 (30.7%)	11 (15.4%)
Malignancy n (%)	29 (26.3%)	11 (28.2%)	18 (25.3%)
Hypertension n (%)	23 (20.9%)	7 (17.94%)	16 (22.5%)
Ischemic heart disease n (%)	8 (7.3%)	3 (7.6%)	5 (7%)
Chronic kidney disease n(%)	9 (8.2%)	4 (10.2%)	5 (7%)
RVI (Retroviral infection) n(%)	11 (10%)	5 (12.8%)	6 (8.4%)
**Illness severity at index admission**			
Septic shock	42 (38.2%)	27 (64.3%)	15(35.7%)
Acute kidney injury	51 (46.4%)	21 (41.2%)	30(58.8%)
Modified Sequential Organ Failure Assessment	4 (2-9)	5 (2-9)	3(2-6)
Charlson score (median)	3 (0-13)	2 (0-13)	4(0-8)
**Antimicrobial data**			
Culture positive	15(13.6%)	3(7.6%)	12(16.9%)
Culture negative	26(23.6%)	10(25.6%)	15(21.1%)
Gram positive	4(3.6%)	1(2.5%)	3(4.2%)
Gram negative	11(10%)	2(5.1%)	9(12.6%)
**Duration of antibiotics**	8 (5-18)	7(3-8)	10(6-19)
**Length of stay in days**	10(7-16)	7.5(4-11)	10(7-20)
**Disposition**			
**Intensive care unit**	28(25.5%)	13(33.3%)	15(21.1%)

### Data completeness

Data were complete for demographic and most clinical variables, with missingness <10% for all except microbiological cultures. Culture results were undocumented in 65 of 110 cases (59.1%), reflecting limited laboratory availability (Table 1).

### Microbiology

**Figure 1:**
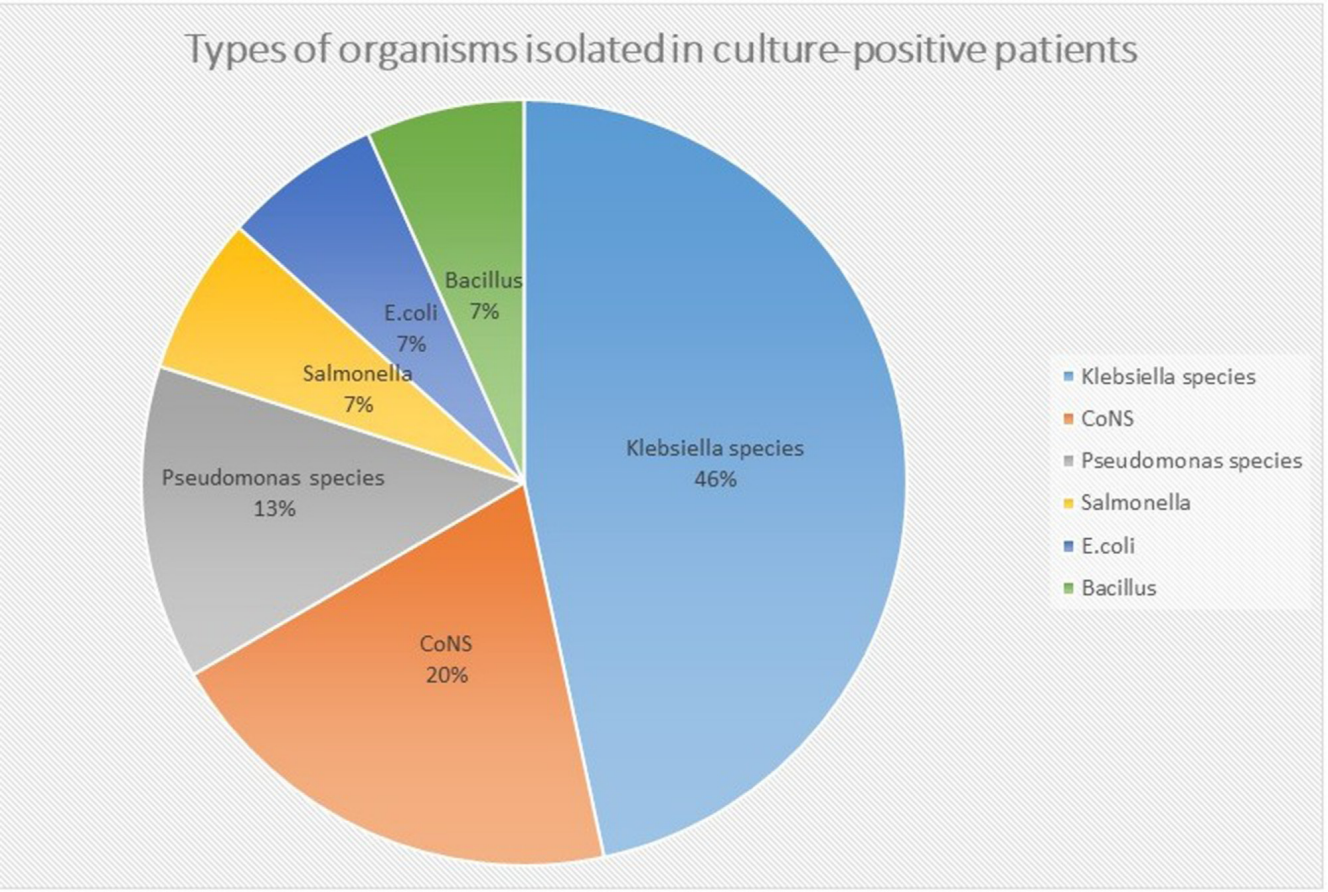
Distribution of organisms isolated from culture-positive sepsis patients (n = 15). Values are numbers (percentage). Klebsiella species 7 (46.7%); CoNS 3 (20.0%); Pseudomonas species 2 (13.3%); Salmonella spp. 1 (6.7%); Escherichia coli 1 (6.7%); Bacillus spp. 1 (6.7%). Gram-negative organisms predominated. CoNS, coagulase-negative staphylococci.

Among culture-positive cases (n = 15), gram-negative organisms predominated; *Klebsiella* species accounted for seven of 15 isolates (46.7% of positives) (Figure 1). Antibiotic therapy was initiated in all 110 patients (100%). The most commonly used classes were beta-lactams (n = 82, 74.5%), aminoglycosides (n = 43, 39.1%), and metronidazole (n = 28, 25.5%), often in combination. The first antibiotic dose was administered within 1 hour in 52% of patients, within 1-3 hours in 31%, and after >3 hours in 17%. Among the 45 patients with culture results, empiric therapy was concordant with sensitivity findings in 33 (73.3%) cases, although the high proportion of missing culture data (59.1%) limits interpretation.

### In-hospital mortality and predictors

Index-hospital mortality was 35.5% (39/110). On multivariable analysis, higher mSOFA and presence of septic shock were independently associated with increased odds of index mortality (mSOFA adjusted OR [AOR] ≈2.51, 95% CI: 1.51-4.18, *P* <0.001; septic shock AOR ≈ 3.38, 95% CI: 1.18-13.58, *P* ≈0.026). Other bivariate associations included older age, higher Charlson score, and ICU admission (Table 3).

**Table 3:** Bivariate and multivariate logistic analysis of factors affecting mortality in sepsis patients.

Variables	Crude OR (95% CI)	*P*-value	Adjusted OR (95% CI)	*P*-value
**Age**	1.026 (1.004-1.048)	0.022	1.016 (0.979-1.054)	0.405
**Charlson score**	1.1899(1.012-1.396)	0.035	1.113 (0.857-1.486)	0.391
**Diabetes mellitus**	0.413(0.162-1.052)	0.064	1.633 (0.396-6.729)	0.493
**Modified Sequential Organ Failure Assessment**	2.915(1.887-4.505)	<0.001	2.513 (1.510-4.183)	<0.001
**Acute kidney injury**	0.480(0.217-1.063)	0.07	0.779 (0.229-2.653)	0.690
**Septic shock**	0.119 (0.049-0.289)	<0.001	3.379 (1.177-13.580)	0.026
**Intensive care unit admission**	0.777-4.484	0.163	2.842 (0.626-12.946)	0.176
**Length of stay**	0.902-1.005	0.077	0.955 (0.885-1.031)	0.239

CI, confidence interval; OR, odds ratio.

### Readmissions: frequency, timing, and clinical features

Of the 71 survivors, 32 (45.1%) were readmitted to TASH within 180 days of discharge. Most readmissions (78.1% of the 32 readmissions) occurred within the first 30 days after discharge; 15.6% occurred between 30-90 days, and 6.3% between 90-180 days. Readmitted patients had a lower median age (43.5 vs 56 years in non-readmitted survivors) and a higher burden of malignancy (34.3% vs 17.9%). Respiratory infection at index admission was the most common source among those later readmitted (40.6%). The readmitted group had a median index length of stay of 14 days (IQR: 8-24) compared with 10 days (IQR: 7-17) in non-readmitted survivors; median antibiotic duration was 14 vs 8 days (Table 4).

**Table 4:** Demography and clinical characteristics of readmitted patients.

Characteristics N (%)	Readmitted 32	Non readmitted 39
**Demographics**		
Age, year median, interquartile range	43.5(28-60)	56(35-67)
**Sex n (%)**		
Male	18(56.3%)	17(43.9%)
Female	14(43.7%)	22(56.4%)
**Source of infection**		
Respiratory	13(40.6%)	15(39.5%)
Urinary	5(15.6%)	11(8.7%)
Gastrointestinal	8(25%)	7(18.4%)
Others	6 (18.7%)	6(15.3%)
**Comorbidity**		
Diabetes mellitus n (%)	7(21.8%)	4(10.2%)
Presence of malignancy n (%)	11(34.3%)	7(17.9%)
Hypertension n (%)	7(21.8%)	9(25%)
Ischemic heart disease n (%)	2(6.2%)	3(8.3%)
Chronic kidney disease n (%)	3(9.3%)	2(5.1%)
RVI n (%)	5(15.6%)	1(3.1%)
**Illness severity**		
Septic shock	8(25%)	9(23%)
Acute kidney injury	12(37.5%)	17(44.7%)
Modified Sequential Organ Failure Assessment	3(2-6)	3(2-5)
**Antimicrobial data**		
Culture positive	7(21.8%)	5(12.8%)
Culture negative	9(28.1%)	7(19.4%)
Gram positive	3(9.3%)	0
Gram negative	4(12.5%)	5(12.8%)
**Duration of antibiotics n (%)**	14(7-24)	8(5-18)
**Length of stay in days n (%)**	14(8-24)	10(7-17)
**Intensive care unit admission at index hospitalization n (%)**	7(21.9%)	8(21.1%)
**Discharge hemoglobin n (%)**	9.5 (6.5-16.4)	10.7(5-18)

### Readmission diagnoses and outcomes

**Figure 2:**
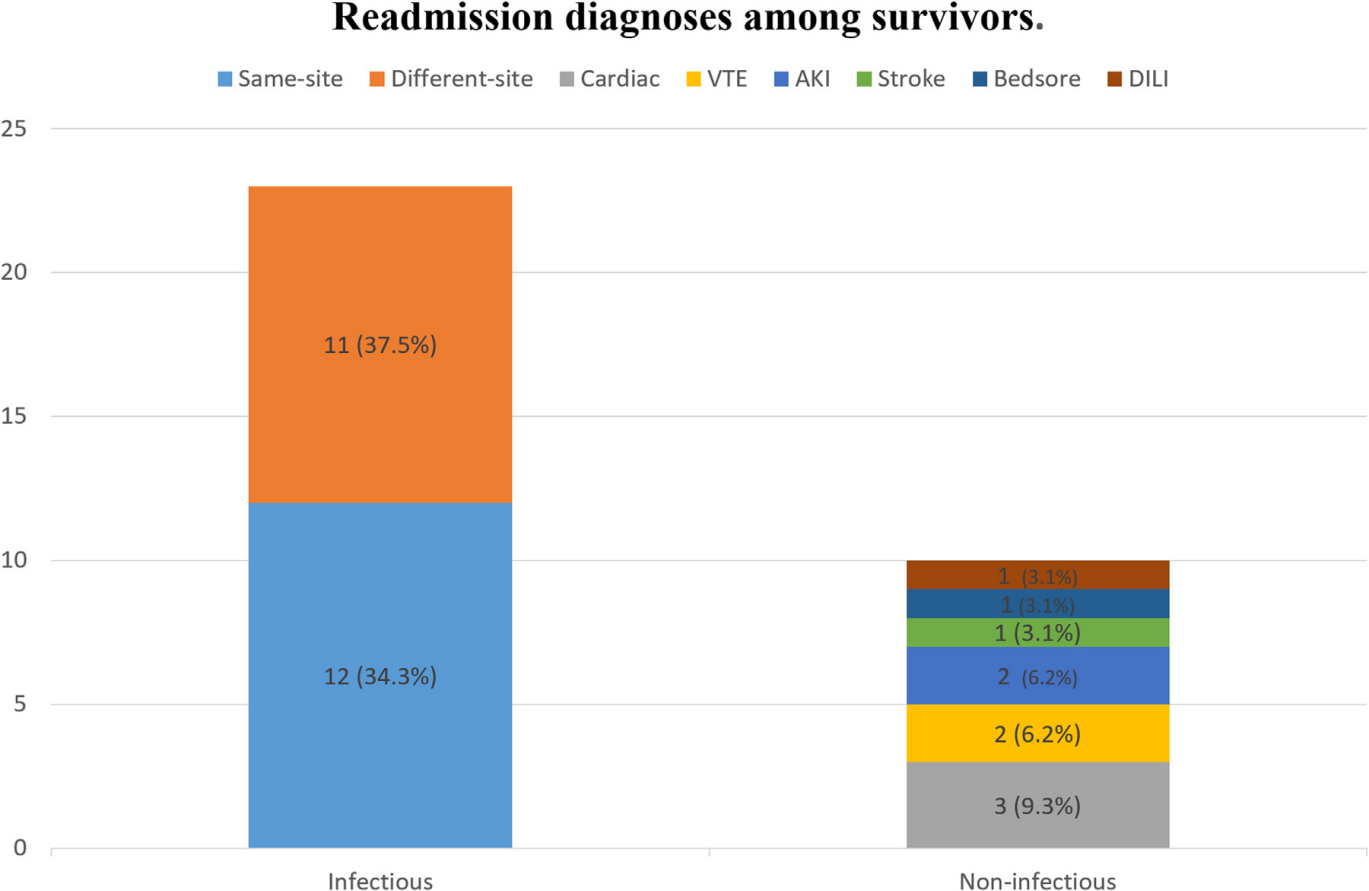
Readmission diagnoses among sepsis survivors (n = 32). Values are numbers (percentage). Infectious causes predominated (23/32, 71.9%), comprising same-site infections 12 (37.5%) and different-site infections 11 (34.4%). Non-infectious causes accounted for 9 (28.1%) and are shown broken down as: cardiac events 3 (9.4%), VTE 2 (6.3%), AKI 2 (6.3%), stroke 1 (3.1%), bedsore 1 (3.1%), and DILI 1 (3.1%). Each bar segment is labeled with n (%) on the figure. AKI, acute kidney injury; DILI, drug-induced liver injury; VTE, venous thromboembolism.

Infections accounted for 71.8% (23/32) of readmissions; 37.5% of readmissions were for the *same-site* infection, and 34.3% for a *different site*. Non-infectious causes comprised 28.2% of readmissions; cardiac events (9.3%), venous thromboembolism (6.2%), and acute kidney injury (6.2%) were the commonest non-infectious diagnoses (Figure 2). Mortality during readmission was 18.7%.

### Predictors of 180-day readmission

**Figure 3:**
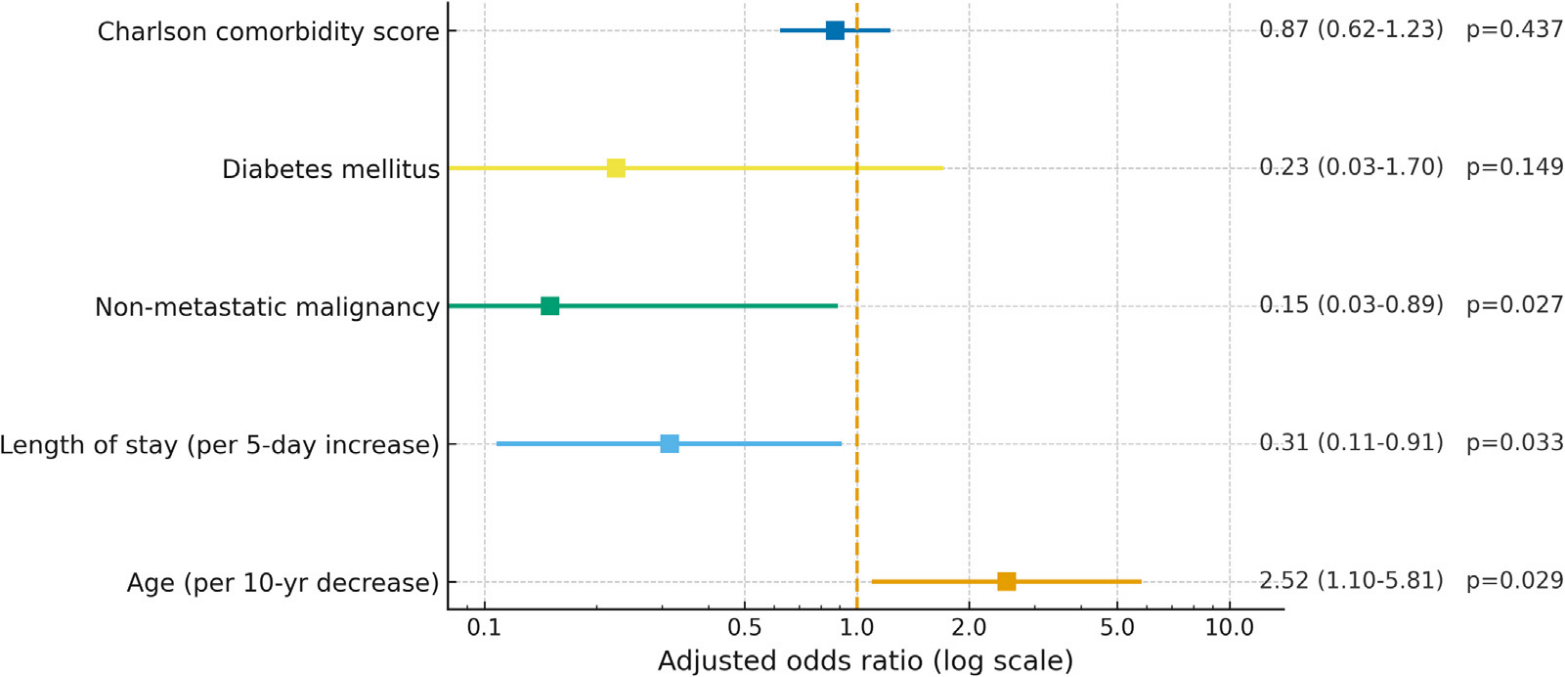
Forest plot of multivariate logistic regression analysis showing factors associated with hospital readmission. Squares represent adjusted odds ratios, and horizontal lines indicate 95% confidence intervals on a logarithmic scale. The dashed vertical line represents the null value (adjusted odds ratio = 1). Age is expressed per 10-year decrease, and length of stay per 5-day increase.

Variables entered into multivariable logistic regression (age, malignancy, length of stay, diabetes, septic shock, acute kidney injury [AKI], mSOFA, Charlson score) produced a model with acceptable fit (Hosmer–Lemeshow *P* =0.356; Nagelkerke R² ≈ 36%) (Table 5). On adjusted analysis, younger age (AOR: 2.53; 95% CI: 1.10-5.81; *P* =0.029), index length of stay (AOR: 0.31; 95% CI: 0.11-0.91; *P* =0.033), and presence of non-metastatic malignancy (AOR: 0.15; 95% CI: 0.03-0.89; *P* =0.027) were independently associated with 180-day readmission (Figure 3).

**Table 5:** Binary and multivariate logistic regression for factors affecting 180-day readmission.

Variables	Crude odds ratio (95% confidence interval)	*P*-value
Age	1.526 (0.896-2.597)	0.120
Presence of malignancy	2.395 (0.800-7.164)	0.118
Length of stay	0.425 (0.189-0.953)	0.038
Septic shock	0.894 (0.275-2.910)	0.853
Diabetes mellitus	2.450 (0.647-9.276)	0.187
Acute kidney injury	1.527 (0.52-4.149)	0.406
Charlson comorbidity score	0.885 (0.736-1.065)	0.196
Modified SOFA score	0.630 (0.230-1.725)	0.369

## Discussion

In this Ethiopian cohort of ED-treated sepsis, 45.1% of hospital survivors were readmitted within 180 days, and most (78.1%) of those occurred within 30 days. These rates exceed those reported from many HIC cohorts [10,12]. Our 30-day readmission (∼35.2%) was markedly higher. Factors likely include the burden of infectious disease and limited outpatient care. The pattern of timing, most readmissions in the first month, aligns with other studies, underscoring an early vulnerability window [9,10].

Infectious causes predominated among readmissions (71.8% of events), consistent with prior literature [9,22]. Pneumonia and urinary tract infections were especially common, mirroring patterns seen in HIC studies (pneumonia often leading to high-risk readmissions) [8,22]. Notably, 37.5% of our infectious readmissions appeared to be recurrent or persistent infections at the same site (e.g., unresolved pneumonia), while 34.3% involved new infection sites. The remainder of readmissions were non-infectious (28.2%), including cardiovascular events (e.g., heart failure, myocardial infarction, stroke), thromboembolic events, and renal failure. This reflects the concept of post-sepsis syndrome, where systemic inflammation and coagulopathy heighten long-term risks of vascular events [11,23]. Indeed, Inghammar et al. [11] found that among Swedish sepsis survivors, the most frequent causes of death and readmission were infection, cancer, and cardiovascular disease. Our mortality during readmission was high (18.7%), underscoring the poor prognosis of these events. Overall, infectious recurrences and new infections drive much of the rehospitalization burden, reinforcing the need for vigilance in managing residual infection and preventing nosocomial pathogens [10,22].

Comparisons with similar low- and middle-income settings are limited but informative. A retrospective cohort study from a tertiary hospital in Pakistan reported a 180-day readmission rate of 31% among sepsis survivors, with infections accounting for approximately two-thirds of readmissions [24]. Regional reviews from SSA document high sepsis mortality and an infectious-disease-dominant etiology (notably HIV and tuberculosis) [1,25], and highlight the paucity of data on long-term outcomes [25]. Differences between our findings and HIC cohorts likely reflect heterogeneity in case mix, higher prevalence of endemic infections, limited microbiology and follow-up infrastructure, and variable access to outpatient care [26]. These contextual differences underscore the need for LMIC-specific strategies for discharge planning, early follow-up, and antimicrobial stewardship.

Predictor analysis yielded several notable findings. Younger age was associated with higher readmission odds, which contrasts with many reports of older age as a risk factor [8,27]. This may reflect a survivor bias: older patients had higher in-hospital mortality (35.5%), leaving fewer older survivors at risk for readmission [27,28]. Conversely, our younger survivors may have had different exposure risks or healthcare-seeking behavior. Non-metastatic malignancy was surprisingly protective (AOR: 0.15), likely because patients with active cancer may have had closer follow-up or because those with less advanced cancer survived. Prior studies typically show cancer is a risk factor for infection and readmission [29], but our finding might be due to excluding metastatic cases or to aggressive in-hospital treatment of known cancers. A longer index hospital stay also reduced readmission odds (AOR: 0.31 per 5-day increase). This may indicate that more complete initial treatment and stabilization mitigated relapse. In contrast, some HIC studies suggest shorter stays are linked to readmission (perhaps due to premature discharge) [8,30].

We did not find septic shock, mSOFA, or ICU admission to be independent predictors after adjustment, although these severity markers are well known to increase mortality risk [1,6]. It is possible that our sample size limited the power to detect all associations. Notably, culture positivity was low (13.6%), reflecting resource limitations. We did not find culture results to predict readmission, but unmeasured antimicrobial resistance or nosocomial pathogens could have influenced outcomes [26]. Our antibiotic duration finding (median 14 days in readmitted vs. 8 in non-readmitted) was not tested in multivariable analysis but suggests a need for judicious stewardship: overly brief courses may lead to relapse, whereas overly long courses can select resistance [14].

Clinically, our results suggest the urgent need for post-discharge interventions for sepsis survivors in Ethiopia and similar LMICs [31]. Discharge planning should recognize that patients with sepsis are at very high risk of early complications; scheduling timely follow-up visits or phone check-ins within the first 2-4 weeks could identify relapses. Indeed, systematic reviews in other diseases show that post-discharge follow-up can reduce 30-day readmissions by about 20% [19,32]. Although specific sepsis follow-up clinics are rare in LMICs, models from HICs, such as post-ICU clinics and rehabilitation programs, aim to address physical and mental impairments [[33], [34], [35]]. Implementing even basic education (warning signs of infection recurrence, medication review, and mobility exercises) may benefit survivors [32]. Antimicrobial stewardship is also key: ensuring appropriate empiric antibiotics and duration could prevent relapses and resistance [36]. Our data suggest that targeting interventions to high-risk subgroups (e.g., younger patients, those with complex infections or chronic illnesses) might be efficient [31].

Prognostically, early readmissions (within 30 days) likely reflect unresolved or recurring infection and may signal the need for aggressive outpatient management [32,37]. Late readmissions (beyond 90 days), although few in our cohort, could represent chronic organ sequelae or new disease processes [11,23]. Recurrent infections raise concern for underlying immunosuppression or resistant organisms [38], while non-infectious events (e.g., cardiovascular) highlight sepsis as a trigger for accelerated chronic disease [39]. Inghammar et al. [11] demonstrated that sepsis survivors have substantially increased mortality out to 5+ years, emphasizing that sepsis is a sentinel event marking vulnerability. Policymakers in Ethiopia should note that sepsis survivors contribute to healthcare utilization long after discharge, underscoring the importance of strengthening emergency and critical care systems [2,4]. The World Health Organization and local health authorities have increasingly recognized sepsis as a global priority; our findings support expanding this to include post-discharge care and survivorship [31,40]. As Lewis et al. note, sepsis burden is likely underestimated in low-income countries, and the hidden cost of readmissions and long-term disability should be addressed in health planning [4].

However, we were unable to assess patients’ post-discharge quality of life, functional recovery, or barriers to follow-up, as these measures were not routinely recorded. These factors likely influence readmission risk and long-term outcomes, particularly in resource-limited settings. Future prospective studies should incorporate brief HRQoL (Health-Related Quality of Life) and functional assessments and structured evaluation of follow-up barriers to better identify modifiable targets for intervention.

### Limitations

This retrospective, single-center study relied on routine chart documentation, which may have caused misclassification and missing values and may limit generalizability. Our adapted mSOFA, while previously used in ICU settings, may imperfectly capture organ dysfunction compared with full SOFA/Sepsis-3. Readmissions were ascertained only at our hospital, so admissions elsewhere or deaths outside the hospital were not captured, likely underestimating true readmission and mortality. Microbiological data were incomplete (blood culture positivity: 13.6%) because of limited laboratory capacity, which may have missed atypical pathogens or resistance patterns. The effective sample for predictor analysis was modest (110 patients, 71 survivors), reducing power and precision. Finally, several potential confounders were unavailable in charts (socioeconomic and nutritional status, structured post-discharge follow-up, patient-reported outcomes, and discharge functional status), and we could not evaluate access barriers to follow-up; these omissions limit causal interpretation and argue for prospective cohorts that collect these data. Despite these constraints, our findings align with international observations and highlight important challenges for post-sepsis care in this setting.

## Conclusion

In this single-center LMIC study, post-sepsis readmissions were alarmingly common: nearly half of survivors were rehospitalized within 6 months, mostly in the first month. Infections, often at the same site as the index event, were the predominant cause, and predictors of readmission included younger age, comorbidity profile, and longer index stay. These findings expose a critical gap in sepsis care in low-resource settings. Enhanced discharge planning, careful antimicrobial management, and close early follow-up should be priorities. Recognizing sepsis as a chronic condition in policy and strengthening post-discharge continuity of care through early follow-up, patient education, and targeted support for high-risk survivors may reduce preventable readmissions and improve long-term outcomes. Prospective studies that integrate brief functional and HRQoL measures with assessments of follow-up access are needed to guide feasible, targeted post-discharge interventions.

## Funding

This research did not receive any specific grant from funding agencies in the public, commercial, or not-for-profit sectors.

## Ethics approval and consent to participate

Ethical approval for this study was obtained from the Institutional Review Board of Addis Ababa University College of Health Sciences (IRB approval no. AAU-CHS/MS/2024/201), with additional permission from the Addis Ababa Health Bureau. Given the retrospective, de-identified design, the requirement for individual informed consent was waived by the IRB. All procedures were conducted in accordance with institutional and national ethical guidelines and the Declaration of Helsinki. Data were anonymized using numeric identifiers and stored securely on password-protected drives.

## Author contributions

MHB and CTM conceptualized the study. MHB, CTM, BTM, MGT, and TW developed the study methodology. MHB, CTM, BTM, NBF, and GBT conducted data curation and formal analysis. MHB, TKA, NBF, and GBT carried out the investigation and validation. MHB, CTM, BTM, TKA, and NBF drafted the manuscript. CTM and MGT critically reviewed and revised the manuscript. FD and TW oversaw project administration and supervision.

## Consent for publication

Not applicable.

## Availability of data and material

The datasets generated and/or analyzed during the current study are available from the corresponding author upon reasonable request.

## Declaration of competing interest

The authors have no competing interests to declare.
